# Heterohybridomas producing human immunoglobulin light chains using CD138-selected bone marrow cells

**DOI:** 10.1016/j.bbrep.2025.102017

**Published:** 2025-04-23

**Authors:** P. Zhou, X. Ma, S. Scalia, D. Toskic, X. Wu, T. Fogaren, Nancy Coady Lyons, Luis del Pozo-Yauner, R.L. Comenzo

**Affiliations:** a Tufts Medicine Myeloma and Amyloid Program, USA; b Division of Hematology-Oncology, Department of Medicine, Tufts Medical Center, Boston, MA, USA; c Department of Pathology, University of South Alabama, Mobile, AL, USA

**Keywords:** Immunoglobulin light chains, Heterohybridomas, CD138, Plasma cells

## Abstract

**Background:**

Light chain research is hampered by lack of mammalian cell lines producing human light chains (FLC). Therefore, we used heterohybridoma (HH) technology to produce clones making FLC thereby providing tools to study light chain behavior.

**Methods:**

Marrow CD138+ cells from patients with multiple myeloma (MM) and polyclonal gammopathy (PG) were selected, fused with B5-6 T cells and cultured in hypoxanthine-aminopterin-thymidine medium (HAT). HH clones were selected based on ELISA for human immunoglobulins and flow cytometry for intracellular (IC) FLC. We compared marrow cell counts and HH yields by diagnosis, evaluated clones making only FLC by flow and by dimer/monomer (D/M) ratios in vitro and in vivo, and sequenced FLC genes with RT-PCR.

**Results:**

Marrows from 13 patients with active disease, 10 MM and 3 PG, were no different in mononuclear or CD138-selected cell counts. HH FLC clones (7 λ, 1 κ) were obtained from 5/10 MM and 2/3 PG; one PG case produced 2 HH FLC clones (one λ and one κ). Of the 10 MM cases, 8 had high risk cytogenetic features and 4 of the 8 produced HH clones while of the 3 PG cases 2 had negative cytogenetics and 1 had loss of IgH identified and produced an HH clone. Mononuclear (MNC) and CD138-selected cell numbers were markedly higher in the samples that enabled productive fusions. Median MFI for the 8 HH clones by IC flow for FLC was 9849 (range, 5344–27451) and median percentage of cells IC positive was 88 % (69–95). Medians of in vitro and in vivo FLC production were 47 μg/mL (9–80) per million cells after 2 days of culture and 66.4 μg/mL (16–1100) in NOD-SCID γ (NSG) mice 14 days after intraperitoneal (IP) implants of 2 × 10^6^ HH cells. Dimer/monomer ratio medians were 0.575 (0.08–0.939) in vitro and 0.91 (0.82–2.7) in vivo, values that were correlated (R^2^ = 0.565) by two-tailed paired *t*-test with *P* < 0.05.

**Conclusions:**

B5-6 T HH producing human FLC were obtained from 50 % of MM and PG cases. High numbers of MNC and CD138+ cells enabled productive fusions. The HH clones produced FLC with easily appreciated dimers and monomers in vitro and in vivo. With IP in vivo implants after 2 weeks more dimers were seen than in short term cultures in vitro. These HH clones will be made available for study of FLC metabolism and testing of therapeutics designed to abrogate FLC production or enable FLC clearance in vivo.

## Highlights

• Heterohybridomas with human plasma cells produce large amounts of free light chains.
• These novel heterohybridomas provide important tools to study light chain behavior.
• In vivo engraftment of these heterohybridomas provides new dimensions for research.

## 1 Introduction

The role of human immunoglobulin free light chains (FLC) remains poorly understood. Intact immunoglobulin molecules require the pairing of a heavy chain with a light chain to form a functional structure. In contrast, free light chains (FLC) can be secreted independently without pairing with heavy chains, as they are produced in excess and readily secreted into the bloodstream. Heavy chains, however, cannot be secreted without pairing with light chains due to stringent cellular quality control mechanisms that retain and degrade unpaired heavy chains. Because FLC are secretory competent and are made in excess in normal plasma cells there are measurable levels of κ and λ FLC in circulation [[1], [2], [3]]. While FLC measurements and activities in hematologic malignancies, as well as in autoimmune and infectious disorders, have been studied, the specific roles of FLC in the latter two and in metabolic processes remains unclear [[4], [5], [6], [7], [8], [9], [10], [11], [12], [13]]. Moreover, given the variable region germline gene segments on chromosome 2 (κ) and 22 (λ) and the distinctive differences in utilization of segments in hematologic disorders such as light-chain amyloidosis and chronic lymphocytic leukemia, there continues to be active investigative work uncovering the basis for the skewed distribution from the normal repertoire seen in these diseases [14]. Importantly, therapeutic interventions aimed at selectively inhibiting cellular activities such as translation or secretion of FLC, or extracellular approaches aimed at stabilizing or clearing circulating human FLC and their pathological deposits, lack models for in vivo testing [[15], [16], [17]]. In this report we provide breakthrough tools, B5-6 T cell heterohybridomas producing large amounts of human FLC enabling both in vitro and in vivo study. These heterohybridomas (HH) were created with human marrow CD138-selected clonal plasma cells from myeloma and hypergammaglobulinemia patients. It is important to note that CD138 (Syndecan 1) is expressed on several subsets of plasma cells, not only clonal but also normal long-lived and earlier subsets in the process of differentiating from B cells [18]. The HH clones we have generated are murine in origin and quite amenable to implantation in NOD-SCID γ (NSG) mice, producing both monomeric and dimeric FLC in murine circulation.

## 2 Patients and methods

### 2.1 Patients

Bone marrow aspirate specimens were obtained from patients with multiple myeloma and polyclonal gammopathy who signed informed consent using an institutional review and privacy board approved protocol. All procedures were performed in compliance with relevant laws and institutional guidelines as approved by the Tufts Medical Center Institutional Review Board (IRB) (Tufts IRB #7138, annual approval on April 17, 2024.) Specimens for research use were obtained simultaneously with clinical samples for hematopathology. To select CD138+ cells, mononuclear cells were separated over Ficoll-Paque PLUS (Amersham Pharmacia Biotech; Uppsala, Sweden) and CD138+ cell selection was performed by immunomagnetic separation as previously described [17,[19], [20], [21]]. After CD138-selection, hemocytometer cell counts for number and viability were performed on CD138-selected cells.

### 2.2 Heterohybridoma production

Mouse-human heterohybridoma (HH) cells were produced following the ClonaCellTM-HY kit from Stem Cell Technologies (Catalog #03800; Cambridge, MA). All methods were as described in the protocol, replacing immunized mouse splenocytes with human CD138+ cells. Mouse myeloma B5-6 T cells (PTA-8869, ATCC, Manassas, VA) were fused with CD138+ cells at a 1:1 ratio. B5-6 T cells ectopically express IL-6 and the telomerase reverse transcriptase gene (TERT) [18]. The cell mixture was centrifuged at 400 g for 10 min. Pre-warmed polyethylene glycol (PEG) was slowly added to the cell pellet for 1 min, then medium containing Dulbecco's Modified Eagle Medium (DMEM), fetal bovine serum, Gentamicin, 2-Mercaptoethanol, Phenol red, l-Glutamine and other supplements was added to the fusion mixture and mixed continuously for 4 min. Additional medium was added to the tube and cells were incubated at 37°C for 15 min; then the fusion cell mixture was washed to remove PEG and the cells were resuspended and cells were incubated overnight at 37°C. The following day the cells were resuspended in pre-warmed hypoxanthine-aminopterin-thymidine (HAT) selection medium and split evenly into flat-bottom 96-well tissue culture plates and incubated for 10–14 days. Supernatants from wells with viable cell growth were removed and ELISAs for immunoglobulin and light chain proteins were performed on them. The wells that corresponded to ELISA FLC positive samples were sub-cloned and re-tested by ELISA; single clones were selected and cultured in DMEM medium with 10 % Fetal Bovine Serum (FBS).

### 2.3 ELISA

Heterohybridoma supernatants for ELISA were evaluated in quantitative sandwich ELISA for human IgA, IgG, IgM, κ and λ light chains (Bethyl Laboratories, Montgomery, TX) according to the manufacturers’ instructions.

### 2.4 Flow cytometry

Heterohybridomas stably secreting human light chains based on confirmative serial measurements by ELISA were evaluated for intracellular light chains by flow cytometry. Antibodies were titrated for optimal use and used with appropriate isotype controls and assays performed on a BD Accuri flow cytometer (Becton, Dickinson and Company, Franklin Lakes, NJ). Flow cytometry for intracellular immunoglobulins was performed with Allophycocyanin (APC)- and phycoerythrin (PE)-conjugated anti-human immunoglobulin and light-chain antibodies titrated for optimal use with appropriate isotype controls (eBiosciences, San Diego, CA, USA). Cells were permeabilized with CytoFix/CytoPerm Fixation/Permeabilization kit (BD Pharmingen, Franklin Lakes, NJ), then stained with antibodies and acquired. Mean fluorescence intensity (MFI) in each case was analyzed with FlowJo (Tree Star, Ashland, OR) and computed minus that of isotype control.

### 2.5 Immunoblots and immunoglobulin light chain dimer/monomer ratio

Supernatants of heterohybridoma cultures were collected and concentrated as needed. The sera of NSG mice after IP inoculation with 2x10^6^ cells per mouse were collected after 14 days. For sodium dodecyl sulfate polyacrylamide gel electrophoresis (SDS-PAGE) 1 mm thick mini gels were manually cast using the mini-protean system (Bio-Rad, 1658001FC) using standard protocols. 4–20 % glycine SDS-PAGE gradient gels contained 10 or 15 wells, depending on the number of samples. Equal amounts of proteins were separated under non-reducing denatured condition with SDS–polyacrylamide gel electrophoresis and electro transferred onto PVDF membranes. Immunoblot (IB) was performed as previously described and probed with multi-cross absorbed HRP-conjugated goat anti-human Ig λ− or goat anti human Ig κ-HRP (Thermo Scientific/Invitrogen, Waltham, MA) at optimized dilutions. Signals were detected with enhanced chemiluminescence using ImageQuant LAS 4000 mini (GE Healthcare Life Sciences, Piscataway, NJ, USA); protein signal densitometry of light chains was conducted by using ImageQuant TL 7.0 (GE Healthcare Life Sciences, Piscataway, NJ, USA) software. The dimer to monomer ratio of human λ or κ FLC was calculated and compared based on signal strength of bands in the immunoblots.

### 2.6 FLC *IGVL gene Amplification and sequence analysis*

RNA extraction from HH cells was performed with the RNeasy Plus Mini-Kit (Qiagen; Hilden, Germany) and cDNA synthesized with the ThermoScript RTPCR System (Invitrogen; Carlsbad, CA, USA) [15,16]. Clonal Ig variable region light chain (*IGVL*) genes were identified as previously described, using PCR primers for consensus Cλ or Cκ regions and for Vλ and Vκ subgroups [19,20]. PCR was conducted with Taq DNA Polymerase (Invitrogen) and the amplicons identified and prepared for core lab sequencing with Wizard SV Gel and PCR Clean-UP System (Promega; Madison, WI, USA). Each specimen was subject to multiple amplifications and bands were selected for sequencing from several separate PCR experiments to confirm the reproducibility of the amplified sequence. With each confirmed sequence we then identified the corresponding *IGVL* germline gene in the ImMunoGeneTics database (IMGT, www.imgt.org). The confirmed sequences were compared with light chain sequences available in AL-Base [21].

### 2.7 NOD scid γ (NSG) mice

All animal experiments were approved by the Tufts Medical Center institutional animal care and use committee (Protocol #B2022-23, annual approval on February 6, 2024). Five-week old female NSG mice were obtained from Jackson Laboratories (Bar Harbor, ME, USA). Employing an intraperitoneal (IP) model as previously described, 10^6^ light-chain producing heterohybridoma cells suspended in phosphate buffered saline (PBS) were injected IP. All mice were observed for health irregularities and weighed at least twice weekly. To obtain blood at indicated time points, the mice were anesthetized and we attempted to collect a minimum of 80 μl from the submandibular vein. At sacrifice, we harvested blood and separated serum for freezing at −20°C.

### 2.8 Statistical analysis

We used PRISM (GraphPad V5, San Diego, CA) for descriptive statistics and analyses.

## 3 Results

### 3.1 Patients

We obtained marrow samples from newly diagnosed patients with plasma cell leukemia (PCL = 2), with multiple myeloma requiring therapy (MM = 9) and with polyclonal gammopathy (PG = 3) due to liver disease in 2 and Sjogren's syndrome with cutaneous light-chain amyloidosis in 1. Characteristics of the 7 patients whose CD138+ cells were successfully fused with B5-6 T cells are shown in Table 1 (PCL = 2, MM = 3, PG = 2) as are the designations of the 8 HH clones. The CD138-selected marrow cells of the 66 year-old female with PG due to liver disease produced 2 HH clones, a λ and a κ. The 2 PCL and 2 of the 3 MM patients had high-risk cytogenetics including gain 1q in all cases as did 4 of the 6 MM patients whose fusion attempts were non-productive (3 of the 4 had gain 1q). There was no significant difference in the clonal free light chain levels between those patients with productive and non-productive fusions.

**Table 1:** Patients with Productive Fusions and Heterohybridoma (HH) Clone Names. This table contains the 8 successful heterohybridomas produced with the CD138-selected marrow cells from 14 patients (57 % success rate).

Sex	Age	DX	M-protein	Marrow Plasma Cells	λ FLC (mg/L)	κ FLC (mg/L)	Clinical findings	Cyto-genetics, FISH	HH clone
M	68	PCL	IgG λ + λ LC IgG = 2860 mg/dL	90 % λ-restricted plasma cells	1180	6	3330 blood plasma cells/uL	LOSS OF 1p, GAIN OF 1q, LOSS OF 13q, 14-20 TRANSLOCATION, AND LOSS OF 17p	VG-68
M	70	MM	IgG λ + λ LC IgG = 3494 mg/dL	30–50 % λ-restricted plasma cells	183.5	16.3	CKD 3 A Fib	Amp 1q Trisomy 7 and 9	LS-82
F	66	PG	IgG = 2764 mg/dL	5–8 % polytypic PCs	65.4	44.4	Immune mediated hepatitis	Loss of IgH	PY-23L PY-23K
F	59	PCL	IgA λ + λ LC IgA = 5848 mg/dL	90 % λ-restricted plasma cells	2166	6	17,850 blood plasma cells/uL	Gain 1q Del 1p t (14:20)	JJ-40
F	71	PG	IgG = 1132 mg/dL IgA = 542 mg/dL	5 % polytypic PCs	27.1	57.8	Fatty liver w/history of EtOH misuse	Negative	MW-13
F	66	MM	IgM biclonal IgM = 631 mg/dL	8 % κ-restricted plasma cells	18.7	230.6	Multiple Comp fx	Negative	DD-91
F	78	MM	Biclonal IgG λ + IgAλ + λ LC	80 % λ-restricted plasma cells	4381	20	Multiple Comp fx	Gain 1q Del 13q Trisomy 9 and 15	WK-54

**CKD** chronic kidney disease. **A fib** atrial fibrillation. **Comp fx** spinal compression fracture.Table.

### 3.2 Cell selection data

Patients with productive fusions had bone marrow MNC that were markedly higher than those of patients with non-productive fusions with medians of 50 × 10^6^ (IQR 30–109) and 18.4 × 10^6^ (13.3–24.7) respectively (*P* < 0.05, Mann Whitney two-tailed). CD138-selected cells were higher from productive cases also with medians of 10 × 10^5^ (IQR 7.5–60) and 3 × 10^5^ (1.5–6.5) respectively (*P* = 0.055, Mann Whitney two-tailed). In all cases CD138+ cells had >90 % viability prior to initiating fusion with B5-6 T cells at a 1:1 ratio.

### 3.3 HH clone evaluations

We evaluated the cells that proliferated after fusion with intracellular flow cytometry for immunoglobulin light and heavy chains. We also evaluated their supernatants for heavy and light chains also. In Fig. 1 we show representative plots depicting the results of a flow cytometry evaluation with intracellular staining for heavy and light chain proteins and in Table 2 the results of FLC in vitro and in vivo production in clones making only FLC, the *IGVL* germlines of the variable region genes captured in each clone, and the Genbank accession numbers for the nucleotide sequence of each clonal gene. In Fig. 2 we show the Western blots of in vitro supernatants displaying the monomer and dimer bands for each HH clone. The variable levels of dimer:monomer ratios both in vitro and in vivo are of particular interest; there was a trend toward significance in the analysis of their relationship (P = 0.09, paired *t*-test two-tailed).

**Fig. 1:**
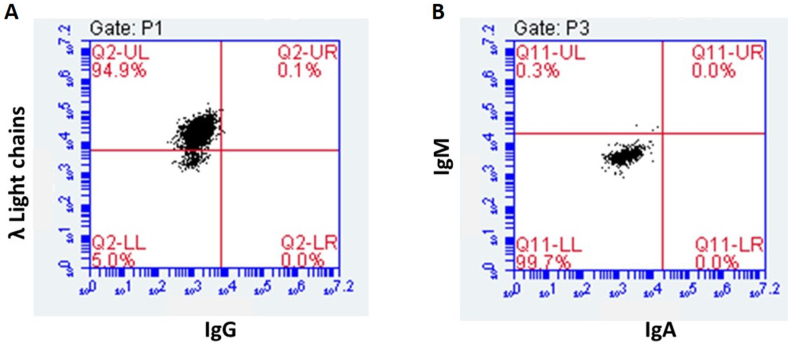
Flow Plots Showing Intracellular Staining of an HH clone for Heavy and Light Chain Immunoglobulins. These representative plots show intracellular staining of a productive HH clone that made λ light chains only and did not make IgG, IgM or IgA heavy chains. This result confirmed the ELISA result showing λ light chains in the supernatant of this clone. We performed flow cytometry and ELISA in order to confirm that the clones we selected made only free light chains.

**Table 2:** Characteristics of the HH Clones: Names, IGVL Germline Genes, Percent Positive by Flow, Production and Dimer:Monomer Data, and Genbank Accession Numbers. The germline donors are identified by putting the nucleotide sequence of the FLC gene into the IMGT/V-QUEST tool. The percentage of cells that have intracellular (IC) staining for the secreted light chain is determined by flow cytometry as shown in Fig. 1A in the upper left-hand quadrant. Median MFI for the 8 HH clones by IC flow for FLC was 9849 (range, 5344–27451) and median percentage of cells that were IC positive was 88 % (69–95). The production of light chains in vitro by 10^6^ cells over 2 days and in vivo with intraperitoneal (IP) implants in NSG mice at 2 weeks is measured in μg/ml of supernatant or mouse serum. The in vitro D:M ratio is estimated by Western blot as shown in Fig. 2 below. D/M ratio medians were 0.575 (0.08–0.939) in vitro and 0.91 (0.82–2.7) in vivo, values that were correlated (R^2^ = 0.565) by two-tailed paired *t*-test with *P* < 0.05. The in vivo D:M ratio in all HH was higher than that in vitro, possibly reflecting a more oxidative environment in a stressed in vivo HH. The gene sequences for the light chains made by these 8 HH clones are in Genbank with accession numbers as shown in the last column.

CLONE	Dx	*IGVL*	%HH^FLC+^	μg/10^6/2d^	D/M^Vitro^	μg/mL/NSG^14d^	D/M^IPVivo^	Genbank #
VG-68	PCL	*LV3-25*	86	77.16	0.939	57.5	0.90	PP112601
LS-82	MM	*LV3-21*	74	80.11	0.582	15.91	1.75	PP196641
PY-23L	PG	*LV3-19*	94	22.65	0.152	38.5	0.91	PP112598
JJ-40	PCL	*LV2-14*	83	46.09	0.564	75.38	0.82	PP112599
MW-13	PG	*LV1-40*	92	30.42	0,567	35.6	0.91	PP196642
DD-91	MM	*LV1-47*	90	47.97	0.650	1100	2.67	PP112597
WK-54	MM	*LV1-47*	95	59.73	0.775	130.87	1.08	PP112600
PY-23K	PG	*KV1-5*	69	8.90	0.085	77	0.85	PP196643

**Fig. 2:**
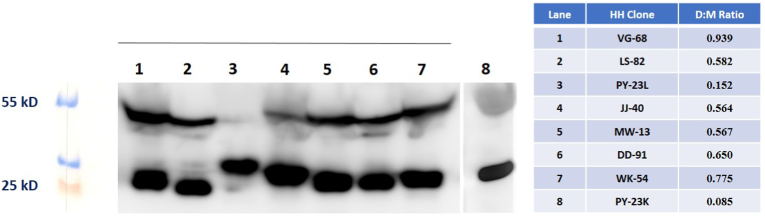
Immunoblots for Dimer:Monomer (D:M) Ratios. Dimers are about 55kD and monomers 25kD. Densitometry was used to provide estimates of the D:M ratios. (For replicate immunoblots see Supplemental Fig. 1).

### 3.4 Sequence analyses

All eight HH clones produced FLC whose germline donors were identifiable with the IMGT tool. Based on analyses in AL-Base, 5 of the 7 clones producing λ FLC were derived from germline donors found predominantly in non-AL plasma cell disorders, *LV 1–40, LV 1–47, LV 3–21, LV 3–25*, and *KV 1–5*, while 2 were derived from germline donors found predominantly in AL, *LV 2–14* and *LV 3–19* [22].

## 4 Discussion

Hybridoma and heterohybridoma technology has historically been focused on the production of intact antibodies to a variety of antigens such as human cell surface antigens often with the intent of producing antibodies for diagnostic or research purposes [[23], [24], [25]].

Novel mouse models and numerous therapeutic applications have been developed of antibody-related immunotherapy not only for hematologic malignancies and solid tumors but also for autoimmune and viral diseases [[26], [27], [28], [29], [30], [31]]. Significant technological advances have enabled commercial discoveries and manipulations of immunoglobulin genes from several species for novel antibody configurations [[32], [33], [34]]. Nevertheless, our knowledge of the behavior of human free light chains remains limited. Their characterization, particularly in vivo, requires cellular models producing light chains [35]. The diversity and complexity of light chain proteins both structurally and behaviorally as vectors of disease as is the case in light-chain amyloidosis (AL) or deposition disease has challenged the development of model systems to detail the stages and phases of seeding and pathologic initiation in organs such as the kidneys and the heart [36,37]. While the role of germline-related factors has been studied productively, it has had limited impact on the development of model experimental systems [[38], [39], [40]]. Research tools producing human light chains such as these heterohybridoma cells are needed so that light chain behavior and potential therapies can be studied in animal models.

In the work we report we chose to use human antibody-producing cells from patients with the clonal plasma cell disorders plasma cell leukemia (PCL), multiple myeloma (MM) and polyclonal gammopathy (PG). With a sample size of only 13 patients, we obtained 8 HH light-chain producing clones, a success rate of 62 %, albeit with careful screening to confirm that the HH clones produced only light-chains. The 13 patients included PCL and MM patients whose plasma cells produced only clonal light chains and PG patients whose B-cell and plasma cell activity produced a range of immunoglobulins. Of note, a PG patient had CD138-selected cells that productively fused to produce two HH clones, one making λ and a second making κ light chains only. Marrow CD138-selected cells in PG patients likely contain a spectrum of CD138+ cells, possibly including some that made light chains only [41]. The selection marker CD138 is expressed on both B-cells differentiating to plasma cells and cloncal plasma cells also [42].

Twenty percent of myeloma patients have clones that produce only light chains; therefore, the opportunity exists to use this technology to make light-chain producing heterohybridomas that, for example, might generate cast nephropathy in mice [43]. We did attempt to make HH clones with CD138-selected cells from patients with AL but failed, in part because the number of CD138+ cells was low and in part because of the possibility that PEG enabled amyloid fibrils to form impairing fused cell viability (data not shown) [44,45]. Additional groups that have active plasma cell subsets include patients with autoimmune diseases and viral illnesses [46,47]. The utilization and mutational patterns for variable region genes, both heavy and light, in these groups are aspects of adaptive immunity that have not been well studied [48,49]. Roles for light chains in some groups, such as Lupus patients, are now, however, being investigated more closely [50,51].

Free light chains can be secreted as dimers or monomers in different proportions, a feature that may be clinically relevant but again has not been well studied [52]. λ light chains are usually dimeric and κ monomeric, a difference that affects the ratio of the two in circulation because monomers are more facilely cleared by the kidneys [53]. In AL dimers appear to play an important role possibly because they are less stable and dissociate more frequently [54,55]. In the 8 HH clones we report, the variability among clones and between in vitro and in vivo values are notable; they invite, for example, in vivo assessment of therapies aimed at stabilizing dimers [16,56]. B5-6 T cells are murine in origin and engraft reliably in immunocompromised mice and human light chains can be reliably measured by ELISA in the serum of these mice as we have shown [57].

In conclusion we offer both a series of HH clones and a method for further development of HH clones producing human free light chains. Our goal is to enable in vitro and in vivo study of human light chain behavior in immunocompromised mice. A better understanding of restrictive light chain repertoires that may be population, disease or infection specific depends on experimental analyses of their behavior, an effort that requires light chain reagents in culture and in vivo. In addition, disorders that occur due to pathologic free light chain monomers may benefit from therapies that clear monomers or stabilize dimers, efforts that also require reagents such as those we report.

## CRediT authorship contribution statement

**P. Zhou:** Methodology, Data curation. **X. Ma:** Methodology, Data curation. **S. Scalia:** Methodology, Data curation. **D. Toskic:** Writing – review & editing, Project administration. **X. Wu:** Resources. **T. Fogaren:** Resources. **Nancy Coady Lyons:** Resources. **Luis del Pozo-Yauner:** Writing – review & editing, Writing – original draft. **R.L. Comenzo:** Writing – review & editing, Writing – original draft, Visualization, Supervision, Formal analysis, Conceptualization.

## Funding sources

This work was supported by 10.13039/100000002NIH/National Institute of Aging grant R21-AG070502 (RLC), by R01-CA279808 (RLC) and by the Janssen QuickFire Challenge Idea Grant. For their continued support of the Tufts Medicine Myeloma and Amyloid Research Fund we thank all of the donors including the Sidewater Family Fund, the Amyloidosis Foundation, David and Barbara Levine (in memoriam), and the Demarest Lloyd Jr Foundation. We also thank the patients and their families across the USA and the clinical research coordinators who contributed to this study.

## Declaration of competing interest

We have nothing to declare.

## Figures and Tables

**figs1:**
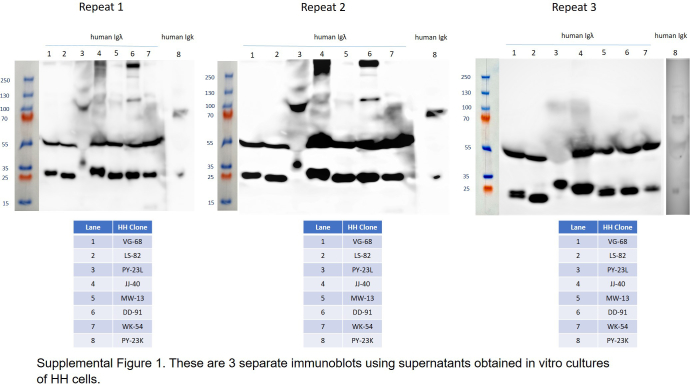


## Data Availability

Data will be made available on request.
