# Supplementary material for: Heterohybridomas producing human immunoglobulin light chains using CD138-selected bone marrow cells
Source: Biochem Biophys Rep. 2025 Apr 23;42:102017. doi: 10.1016/j.bbrep.2025.102017 (PMC12051113; doi:10.1016/j.bbrep.2025.102017)
Supplement: Multimedia component 1 [file mmc1.docx]

| Clone | CDR1 | CDR2 | CDR3 |
| --- | --- | --- | --- |
| VG-68 | 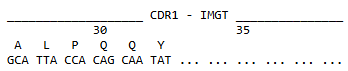 | 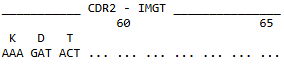 | 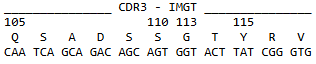 |
| LS-82 | 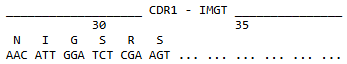 | 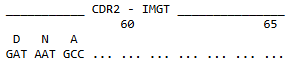 | 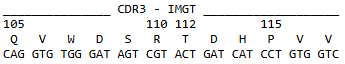 |
| PY-23L | 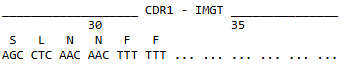 | 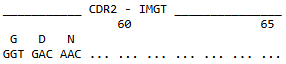 | 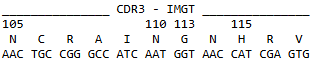 |
| JJ-40 | 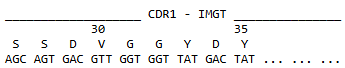 | 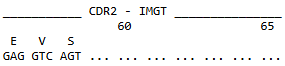 | 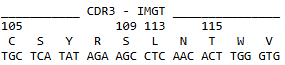 |
| MW-13 | 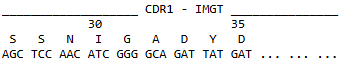 | 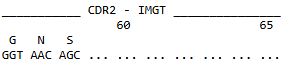 | 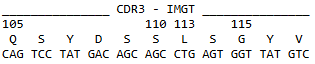 |
| DD-91 | 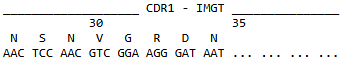 | 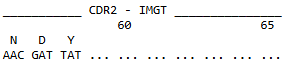 | 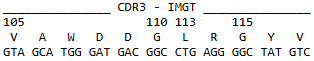 |
| WK-54 | 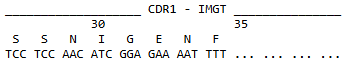 | 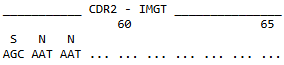 | 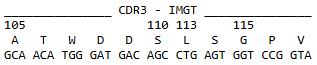 |
| PY-23K | 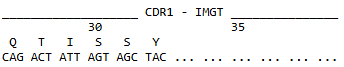 | 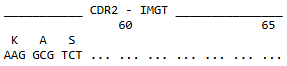 | 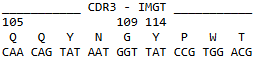 |

**Supplemental Table 1.** CDR regions for each heterohybridoma clone.
